# Incidence and Prediction of Falls in Dementia: A Prospective Study in Older People

**DOI:** 10.1371/journal.pone.0005521

**Published:** 2009-05-13

**Authors:** Louise M. Allan, Clive G. Ballard, Elise N. Rowan, Rose Anne Kenny

**Affiliations:** 1 Institute for Ageing and Health, Wolfson Research Centre, Newcastle General Hospital, Newcastle upon Tyne, United Kingdom; 2 Wolfson Centre for Age Related Disorders, King's College London, London, United Kingdom; 3 Trinity College Institute of Neuroscience, Trinity College Dublin, Dublin, Ireland; James Cook University, Australia

## Abstract

**Background:**

Falls are a major cause of morbidity and mortality in dementia, but there have been no prospective studies of risk factors for falling specific to this patient population, and no successful falls intervention/prevention trials. This prospective study aimed to identify modifiable risk factors for falling in older people with mild to moderate dementia.

**Methods and Findings:**

179 participants aged over 65 years were recruited from outpatient clinics in the UK (38 Alzheimer's disease (AD), 32 Vascular dementia (VAD), 30 Dementia with Lewy bodies (DLB), 40 Parkinson's disease with dementia (PDD), 39 healthy controls). A multifactorial assessment of baseline risk factors was performed and fall diaries were completed prospectively for 12 months. Dementia participants experienced nearly 8 times more incident falls (9118/1000 person-years) than controls (1023/1000 person-years; incidence density ratio: 7.58, 3.11–18.5). In dementia, significant univariate predictors of sustaining at least one fall included diagnosis of Lewy body disorder (proportional hazard ratio (HR) adjusted for age and sex: 3.33, 2.11–5.26), and history of falls in the preceding 12 months (HR: 2.52, 1.52–4.17). In multivariate analyses, significant potentially modifiable predictors were symptomatic orthostatic hypotension (HR: 2.13, 1.19–3.80), autonomic symptom score (HR per point 0–36: 1.055, 1.012–1.099), and Cornell depression score (HR per point 0–40: 1.053, 1.01–1.099). Higher levels of physical activity were protective (HR per point 0–9: 0.827, 0.716–0.956).

**Conclusions:**

The management of symptomatic orthostatic hypotension, autonomic symptoms and depression, and the encouragement of physical activity may provide the core elements for the most fruitful strategy to reduce falls in people with dementia. Randomised controlled trials to assess such a strategy are a priority.

## Introduction

The prevalence of neurodegenerative disorders is increasing due to changes in population demographics. It is estimated that by 2020 there will be 42 million people with a diagnosis of dementia worldwide,[1] in whom the most common causes of dementia will be Alzheimer's disease (AD), Vascular dementia (VAD) and the Lewy body dementias.[2], [3] Falls are a significant cause of injuries, loss of confidence, increased morbidity, institutionalisation and mortality in all older people,[4], [5] but particularly those with dementia.[6], [7] People with dementia recover less well after a fall than those without dementia.[8] In view of the suffering caused by such falls, and the enormous cost of caring for people with dementia who have fallen, there is an urgent need to optimise the prevention of falls in this group.

Importantly, there have been no prospective studies of multifactorial risk factors for falling specific to the population with dementia. Previous studies have identified multiple risk factors for falls in the older population as a whole, in a variety of settings. In multivariate studies significant risk factors include fall history, gait, balance and mobility impairments, visual impairment, cognitive impairment, fear of falling, environmental hazards, muscle weakness and incontinence.[9] In community settings, impairment on various cognitive measures has been shown to be a risk factor for falls, although dementia itself has only been examined and shown to be a risk factor for falls in community dwelling people with Parkinson's disease,[10] and in residents of extended care settings.[7], [11] The identification of potentially modifiable risk factors has been critical in the development of effective multifactorial falls intervention programmes, particularly for older people at high risk of falling.[12] It is likely that falls in dementia are also multi-factorial in origin, possibly with risk factors similar to those identified in the general older population, but there may be other potentially modifiable factors specific to dementia. It is thought that those with Lewy body (LB) dementias are at particular risk, and a history of recurrent falls is accepted as a supporting feature for the diagnosis of dementia with Lewy bodies (DLB).[13]

Only one previous multifactorial intervention study has exclusively recruited participants with dementia.[14] Interventions were based upon those used in successful trials in people without dementia, and the study did not show a significant reduction in falls or number of fallers. However, 70% of the participants in this trial lived in institutional settings, most had moderate to severe cognitive impairment and all had already presented to hospital with a fall. A recent systematic review of interventions to prevent falls in hospitals and care homes suggested that effect size was smaller in trials where the prevalence of dementia was high, but meta-regressions were limited by incomplete reporting of dementia prevalence.[15] It remains possible that multifactorial interventions would be successful in patients with less severe dementia, but we suggest that interventions in future trials should be tailored to potentially modifiable factors which can be shown to predict falls in community dwelling people with mild-moderate dementia. There is therefore an urgent need to understand risk factors for falls in dementia to enable a more tailored and effective intervention to be developed.

Participants in this study completed a multifactorial baseline assessment of putative predictors of falls and then completed prospective falls diaries for a period of 12 months, which is known to be a robust method of ascertainment of falls. We aimed to identify potentially modifiable predictors of falls in older people with mild-moderate dementia, the majority of whom lived in the community.

## Methods

### Ethics statement

This study was approved by the Joint Ethics Committee of Newcastle and North Tyneside Health Authority, the University of Newcastle upon Tyne and the University of Northumbria at Newcastle and participants gave written informed consent in accordance with the declaration of Helsinki.

### Design

Longitudinal cohort study.

### Participant recruitment

We recruited consecutive cases with dementia (AD, VAD, DLB or Parkinson's disease dementia (PDD)) from Neurology, Old Age Psychiatry and Geriatric Medical secondary care outpatient clinics within the Northern Region of the United Kingdom. Cases were referred to these clinics for assessment, diagnosis and management by their primary care physician, or in the case of those with VAD following a stroke, they may have been referred to a Psychiatrist by their stroke physician. We recruited a healthy control group of comparable age by local advertisement.

### Inclusion and exclusion criteria

All participants were over 65 years of age. All diagnoses of dementia were made according to DSM IV criteria. Significant medical causes of dementia were excluded during diagnostic investigations. Diagnoses were made by operationalised criteria for AD,[16] VAD,[17] DLB[18] and PDD,[19] which have been validated against neuropathological diagnosis within our group.[20], [21]

Participants were excluded if they declined participation, died or withdrew from the study before commencing falls diaries, were unable to perform the gait assessments due to other co-morbid conditions, had an MMSE score[22] less than or equal to 8, or were too visually impaired to complete cognitive assessments. Controls were excluded if they had any evidence of dementia or Parkinson's disease.

### Baseline Clinical Assessment

All participants received a detailed baseline assessment to quantify putative risk factors for falls. The factors included were selected on the basis of their previous identification as risk factors in more than one high quality study in older people, and/or their relevance to clinical features of dementia postulated to be causative of falls in dementia.

Assessments included medical history (duration of dementia, residence, history of previous falls and medications). Objective assessments included the cognitive subsection of the CAMDEX (CAMCOG),[23] a physical activity scale previously validated in older people,[24] body mass index, performance-oriented assessment of mobility[25] and the motor subsection of the Unified Parkinson's disease rating scale (UPDRS[26]) to evaluate extra-pyramidal signs. Participants were assessed after taking their usual dose of levodopa, if applicable, and were allowed to use their usual walking aids.

Dementia specific scales were used to assess activities of daily living (Bristol scale[27]), depression (Cornell scale[28], [29]) and behavioural and psychological symptoms of dementia (Neuropsychiatric Inventory[30]), in addition to detailed autonomic assessments as described below. These factors have all been postulated as potential causes of falls in dementia, particularly in the LB dementias.[13]

### Autonomic Assessment

All assessments took place in the morning; participants refrained from consuming caffeinated drinks or smoking on the morning of the assessment. Assessments were carried out according to the protocols described in our previous study.[31] Briefly, blood pressure was monitored using a digital photoplethysmograph (Portapres, TNO, Amsterdam), which enables non-invasive beat-to-beat blood pressure measurement. Orthostatic hypotension (OH) was defined as a fall in systolic blood pressure of greater than 20 mm Hg or diastolic blood pressure of greater than 10 mm Hg that did not return to baseline within 30 seconds from the start of the active stand. Return to baseline was defined as the start of a series of 3 consecutive beats in which the blood pressure was within one standard deviation of the baseline blood pressure. Participants were asked to report symptoms on standing; if dizziness, lightheadedness, unsteadiness or presyncope were reported in the presence of OH this was defined as symptomatic OH. Other clinical autonomic function tests included isometric exercise, Valsalva manoeuvre and deep breathing. Ewing's battery was used to identify the presence of a clinical autonomic neuropathy for each patient who had complied with sufficient tests for the classification scheme to be applied.[32]

### Outcome Measures

The primary outcome measures were prevalence and incidence of falls occurring during the 12 month follow up period. Secondary outcome measures were proportional hazard ratios for time to first fall in dementia, according to diagnosis and status of putative clinical predictors. A fall was defined as an event whereby a person comes to lie on the ground or another lower level with or without loss of consciousness. Participants were given diaries to record the occurrence of falls, to be returned every four weeks in a postage paid envelope. If the participants did not return the diaries they were reminded by telephone after 2 weeks. The caregiver was asked to assist in the completion of diaries when dementia was present.

### Statistics

Differences in baseline characteristics across groups were compared using Fisher's Exact test for categorical data, ANOVA for normally distributed data and Kruskal-Wallis for non-normally distributed data. Differences between individual groups were compared using the Chi squared test for categorical data, Student's t test for normally distributed data and Mann-Whitney U test for non-normally distributed data.

Due to censoring of falls data from some participants (as a result of death or withdrawal from the study), Cox regression (adjusted for age and sex) was used to obtain a proportional hazard ratio for time to first fall in dementia, using healthy controls as the reference group.

The incidence density of falls in each diagnostic group was calculated by the total number of falls in each group per number of person years of diaries returned, expressed as number of falls per 1000 person years. Loglinear Poisson regression models (adjusted for age and sex) were used to obtain an incidence density ratio for dementia and its subtypes in comparison with healthy controls. AD, VAD and DLB were also used as reference groups to examine the effect of dementia subtype upon incidence of falls.

In order to examine the associations between exposure to putative risk factors for falling and the occurrence of falls in those participants with dementia, Cox regression was used to obtain univariate proportional hazard ratios for each risk factor, adjusted for age and sex, using time to the occurrence of at least one fall as the dependent variable. Hazard ratios were given according to presence or absence of the risk factor, or per point on quantitative scales as appropriate. Analyses were performed initially for all participants with dementia, and then repeated stratified by diagnosis.

Following identification in univariate models, significant and potentially modifiable risk factors were entered into a multivariate forward stepwise Cox regression model, p 0.05 for entry, p 0.1 for removal. Age and sex were included even if not significant. Where similar clinical features were described by more than one significant risk factor the factor with the higher level of significance in univariate analyses was entered into multivariate analyses, in order to avoid co-aggregation of predictors; e.g. abnormal pull test rather than the full Tinetti balance scale as both these tests assess balance.

## Results

### Participants

**Figure 1:**
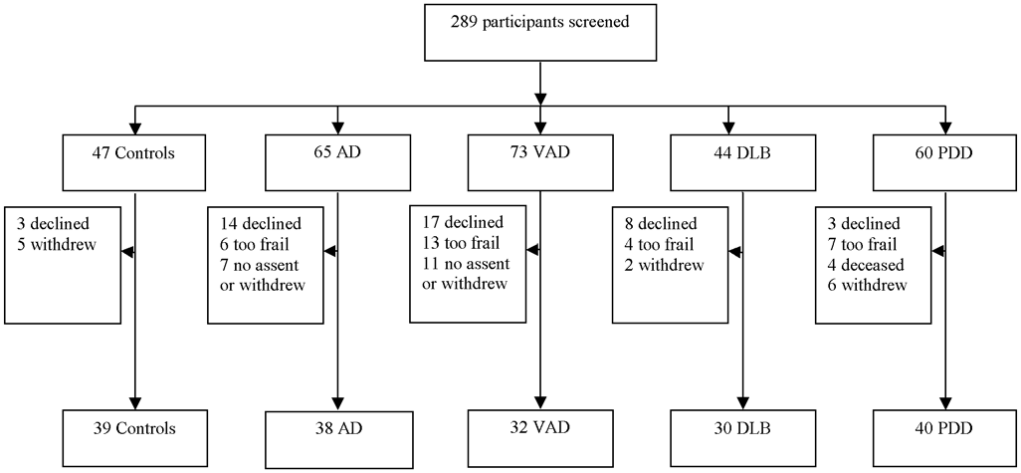
Flow chart to show recruitment of patients to study .

289 patients were considered for inclusion; 65 participants were excluded (Figure 1). 179 (80%) of 224 eligible participants agreed to take part (39 controls, 38 AD, 32 VAD, 30 DLB and 40 PDD). 116 (83%) of 140 participants with dementia and all of the controls were residing in the community. A summary of baseline characteristics of the participants is shown in table 1.

**Table 1:** Baseline characteristics by diagnosis: all participants

Diagnosis (n)	Control (39)	AD (38)	VAD (32)	DLB (30)	PDD (40)
Mean Age (SD)*	75 (6.4)	79 (5.8)	79 (6.2)	76 (7.1)	72 (6.0)
Gender: male (%)	21 (53.8)	18 (47.4)	23 (71.9)	18 (60.0)	26 (65.0)
Median duration of dementia: months (IQR)	-	36 (21–48)	18 (9–30)	24 (12–48)	24 (15–48)
Mean CAMCOG score (SD)**	94 (4.7)	59 (15)	64 (18)	59 (15)	64 (16)
Resident in care home n (%)	0 (0)	6 (15.8)	6 (18.8)	9 (30)	3 (7.5)
History of falls in previous 12 months n (%) ***	13 (33.3)	19 (51.4)	21 (65.6)	18 (69.2)	33 (86.8)
History of recurrent falls in previous 12 months n (%) †	2 (5.1)	10 (26.3)	17 (53.1)	12 (40.0)	29 (72.5)
Uses walking aid or requires assistance to walk n (%) ††	3 (7.7)	5 (13.2)	9 (28.1)	14 (46.7)	27 (67.5)
Cardiovascular medication n (%) †††	19 (48.7)	27 (71.1)	28 (87.5)	20 (66.7)	39 (97.5)
Psychotropic medication n (%) ‡	5 (12.8)	11 (28.9)	19 (59.4)	15 (50.0)	20 (50.0)
Symptomatic orthostatic hypotension n (%) ‡‡	0/39 (0)	2/37 (5.4)	3/31 (9.7)	4/26 (15.4)	12/38 (31.6)

SD: Standard deviation; IQR: Inter-quartile range; AD: Alzheimer's disease; VAD: Vascular dementia; DLB: Dementia with Lewy bodies; PDD: Parkinson's disease dementia; PD: Parkinson's disease. Denominators are given for prevalence (%) where data is incomplete.

* Control vs. patients p = 0.457; Control vs. AD p = 0.036; control vs. VAD p = 0.027; Control vs. DLB p = 0.945; Control vs. PDD p = 0.031.

** Control vs. all patient groups p<0.001. No significant differences between patient groups.

*** Control vs. AD p = 0.087; control vs. VAD p = 0.009; Control vs. DLB p = 0.005; Control vs. PDD p<0.001

† Control vs. AD p = 0.013; control vs. VAD p<0.001; Control vs. DLB p = 0.001; Control vs. PDD p<0.001

†† Control vs. AD p = 0.481; control vs. VAD p<0.024; Control vs. DLB p<0.001; Control vs. PDD p<0.001

††† Control vs. AD p = 0.063; control vs. VAD p = 0.001; Control vs. DLB p = 0.151; Control vs. PDD p<0.001

‡ Control vs. AD p = 0.098; control vs. VAD p<0.001; Control vs. DLB p = 0.001; Control vs. PDD p = 0.001

‡‡ Control vs. AD p = 0.234; control vs. VAD p<0.082; Control vs. DLB p<0.022; Control vs. PDD p<0.001

### Prevalence of falls

**Figure 2:**
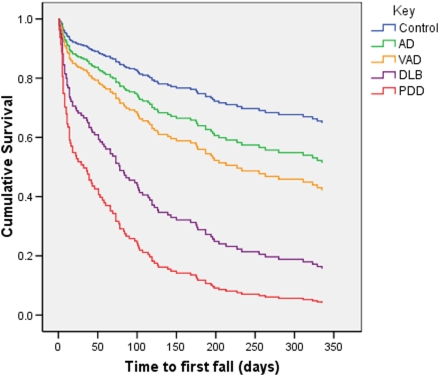
Survival curve showing time to first fall by diagnosis.

81.6% of diaries were returned (82.9% in those participants with dementia). During the 12 month follow up period 65.7% of participants with dementia had at least one fall, compared with 35.9% of controls (relative hazard ratio (HR) adjusted for age and sex: 3.03, 95% confidence intervals (CI) 1.71–5.35). With respect to dementia subtypes, the prevalence of falls in AD was 47%; VAD 47%; DLB 77% and PDD 90%. Figure 2 shows the survival curves until the occurrence of a fall for each diagnosis.

### Incidence of falls

The incidence of falls in dementia was 9118 per 1000 person years, which was significantly higher than in controls (1023; incidence density ratio (IDR) adjusted for age and sex: 7.58, 95% CI: 3.11–18.5), (table 2). If participants living in care homes were excluded, the incidence of falls in dementia was 8763 which remained significantly higher than in controls (IDR: 9.56, 95% CI: 6.07–15.1).

**Table 2:** Annual incidence of falls

	Control (39)	AD (38)	VAD (32)	DLB (30)	PDD (40)
**Incidence**: number of falls/1000 person years	1023	2486	3135	9087	19 000
Incidence density ratio (95% CI) c.f. control group	1	**1.95 (1.01–3.78)**	**1.77 (1.17–2.69)**	**6.06 (3.53–10.4)**	**20.5 (10.4–40.2)**
Incidence density ratio (95% CI) c.f. AD group		1	0.907 (0.504–1.63)	**3.10 (1.16–8.28)**	**10.5 (3.32–33.1)**
Incidence density ratio (95% CI) c.f. VAD group			1	**3.41 (1.96–5.95)**	**11.6 (5.73–23.3**
Incidence density ratio (95% CI) c.f. DLB group				1	**3.38 (2.66–4.31)**
**Fractures**: number of fractures recorded during study	0	1	1	2	3

With respect to dementia subtypes, the incidence of falls, adjusted for age and sex, was higher in all dementia subtypes than in controls (table 2): AD: 2486 falls/1000 person years (IDR: 1.95; 95% CI: 1.01–3.78), VAD: 3135 (IDR: 1.77, 95% CI: 1.17–2.69), DLB: 9087 (IDR: 6.06, 95% CI: 3.53–10.4) and PDD: 19000 (IDR: 20.5, 95% CI: 10.4–40.2). The incidence of falls in PDD was higher than in AD (IDR: 10.5, 95% CI: 3.32–33.1), VAD (IDR: 11.6, 95% CI: 5.73–23.3), and DLB (IDR: 3.38, 95% CI: 2.66–4.31). The incidence of falls in DLB was also higher in AD (IDR: 3.10, 95% CI: 1.16–8.28) and VAD (IDR: 3.41, 95% CI: 1.96–5.95). The incidence of falls in VAD was not significantly higher than in AD (IDR: 0.907, 95% CI: 0.504–1.63).

### Univariate predictors of falls in dementia

Significant modifiable and non-modifiable predictors of falls in all participants with dementia are shown in table 3. Significant predictors included diagnosis of Lewy body disorder, history of falls or recurrent falls in the preceding 12 months, use of cardioactive medication, abnormal gait or balance score, Cornell depression score ≥10, autonomic symptom scale >7, autonomic neuropathy, symptomatic OH and time taken for blood pressure to return to baseline on standing. Age and increased physical activity were protective.

**Table 3:** Univariate predictors of falls in participants with dementia

Diagnosis	n	All participants with dementia		Stratified by diagnosis	
		Relative Hazard ratio	95% confidence intervals	Relative Hazard ratio	95% confidence intervals
**Non modifiable risk factors**					
Age (years)	140	**0.969**	**0.940–0.999**	0.999	0.968–1.03
Male Gender	140	1.08	0.710–1.65	1.04	0.679–1.60
Diagnosis of Lewy Body disorder (PDD or DLB)	140	**3.33**	**2.11–5.26**	-	-
Duration of dementia (per month)	133	1.01	0.998–1.02	**1.02**	**1.00–1.02**
Resident in care home	140	1.57	0.814–3.02	1.76	0.878–3.55
History of falls in previous 12 months	140	**2.52**	**1.52–4.17**	**2.04**	**1.21–3.46**
History of recurrent falls in previous 12 months	140	**2.79**	**1.82–4.29**	**2.28**	**1.44–3.63**
CAMCOG score (0–105, per point)	131	0.999	0.984–1.01	1	0.984–1.02
**Potentially modifiable risk factors**					
Cardioactive medication	140	**2.08**	**1.15–3.75**	**1.91**	**1.03–3.54**
Psychotropic medication	140	1.49	0.986–2.25	1.36	0.885–2.07
Tinetti gait score <7 and/or Tinetti balance score <22	139	**2.12**	**1.39–3.24**	1.14	0.678–1.90
Physical activity score (0–9, per point)	140	**0.818**	**0.723–0.926**	**0.873**	**0.766–0.994**
NPI aberrant motor behaviour sub-score (0–12, per point)	121	0.989	0.922–1.06	0.982	0.916–1.05
Cornell score ≥10	132	**2.01**	**1.18–3.43**	1.26	0.728–2.18
Total autonomic symptom score ≥7	139	**2.27**	**1.49–3.45**	**1.64**	**1.02–2.63**
Definite, atypical or severe autonomic neuropathy	93	**2.01**	**1.16–3.47**	1.24	0.688–2.24
Symptomatic orthostatic hypotension	132	**2.07**	**1.19–3.61**	1.47	0.823–2.62
Time taken for blood pressure to return to baseline on standing (per second)	133	**1**	**1.00–1.01**	**1**	**1.00–1.01**

However, when univariate analyses were stratified by diagnosis, only duration of dementia, history of falls or recurrent falls in the preceding 12 months, use of cardioactive medication, autonomic symptom scale greater than 7 and time taken for blood pressure to return to baseline on standing remained significant predictors of falls. Increased physical activity remained protective.

### Multivariate predictors of falls and recurrent falls in dementia

Significant potentially modifiable predictors were entered into multivariate analyses in the order: age, gender, Tinetti gait score, Cornell depression score, physical activity score, autonomic symptom score, symptomatic orthostatic hypotension, use of cardioactive medication and time for systolic blood pressure to return to baseline on standing.

In the first model including all participants with dementia, predictors retained were Cornell depression score, total autonomic symptom score and symptomatic orthostatic hypotension. In the second model, stratified by diagnosis, predictors retained were symptomatic orthostatic hypotension, use of cardioactive medication and physical activity score, which was protective (Table 4).

**Table 4:** Multivariate predictors of falls in participants with dementia

Diagnosis	All participants with dementia		Stratified by diagnosis	
	Relative Hazard ratio	95% confidence intervals	Relative Hazard ratio	95% confidence intervals
Predictors of falls				
Cornell depression score (0–40, per point)	1.05	1.01–1.10		
Total Autonomic symptom score (0–36, per point)	1.05	1.01–1.10		
Symptomatic orthostatic hypotension	2.13	1.19–3.80	2.2	1.19–4.06
Physical activity score (0–9, per point)			0.827	0.716–0.956
Use of cardioactive medication			1.98	0.994–3.96

## Discussion

In the largest prospective study of predictors of falls in dementia to date, we have demonstrated that older people with dementia experience 8 times more incident falls than those without dementia. These figures are even more striking when only community dwelling people with dementia are considered, with incidence in people with dementia nearly 10 times higher than in those without dementia. Patients with Lewy Body dementias (DLB or PDD) were at the highest risk, with DLB patients sustaining 6 times the number of falls in the control group and PDD 20 times more falls. The annual incidence of falls was higher in the LB dementias than in all other groups studied and much higher than any previous reports in older people[33]. Incidence of falls was higher in PDD than in DLB.

To our knowledge, this is the first study which has identified predictors specific to dementia, including the identification of non-modifiable predictors such as a diagnosis of Lewy body disorder, longer duration of dementia and previous history of falls or recurrent falls. These factors will be useful in identifying individuals at particular risk, who may benefit from further assessment and intervention. Even more importantly, a number of the predictors identified are potentially modifiable, and should be included as key elements of a multifactorial intervention. These factors included use of cardioactive medications, autonomic symptoms, symptomatic orthostatic hypotension, depression and limitation of physical activity. We suggest that interventions targeted towards these predictors could reduce the burden of falls related morbidity and mortality in community dwelling people with mild-moderate dementia.

Our study has a number of strengths which reinforce our findings. This was a prospective study and the majority of participants had mild-moderate dementia and were residing in the community. The incidence of falls was determined by completion of daily diaries, with the support of a caregiver where appropriate, with an adequate follow up period. The use of this method is particularly important because it has the highest sensitivity for accurate recording of falls. The number of participants in our study was high (179); 80% of those approached agreed to take part and compliance with fall diary returns was also high. Statistical analysis was strengthened by the use of Cox regression models and loglinear analysis of incidence densities, thus taking account of censoring of falls diaries in some participants. All analyses included adjustment for age and gender and the inclusion of multivariate analyses enabled efficient estimate of significant associations while adjusting for a number of confounding factors simultaneously.

There are some potential limitations to this study. Ideally, we would have recruited both groups using random sampling from a community population. Unfortunately the scale of such an exercise would have been well beyond the resources available for this study. Healthy older people volunteering for participation in research in response to advertisements are often fitter than the older population at large, but interestingly the incidence of falls in our control group was very similar to that found in most community based studies, suggesting that the control group was reasonably representative, despite the limitations of using this recruitment method. The recruitment of the patient participants from secondary care clinics rather than from primary care may have biased the sample towards those with more progressive disease. However, in the northern region of the UK, suspected cases of Parkinson's disease are routinely referred to a secondary care physician specialising in movement disorders (either a Neurologist or Geriatrician). These patients should, therefore, be reasonably representative of all patients presenting to primary care physicians with symptoms suggestive of PD. There is a greater likelihood of patients with AD or VAD not being referred to secondary care as dementia is often undetected, or not managed by a specialist in memory disorders. The reader should therefore bear in mind that participants in this study may have had more severe or progressive disease than those generally seen in primary care. In interpreting our results, consideration should be given to the need to stratify for dementia subtypes in univariate and multivariate analyses. Some of the predictors were not significant when analyses were stratified by dementia subtype, probably because these predictors were actually surrogate markers of a diagnosis of a Lewy body disorder, which itself predicted falls. In comparison with the proportions in the general population the LB dementias were over represented in our study, in order to make valid comparisons between dementia subtypes.

Prior to commencement of our study we identified only two studies of falls in dementia which included a fully multifactorial assessment of risk factors, the first of which had an inadequate follow up period,[34] and in the second the primary outcome was fall related serious injuries rather than falls.[35] Both of those studies included fewer than 100 participants, and neither included a control group. Two studies published since we commenced our study have included multifactorial baseline assessments, but the first of these was small (only 42 participants with advanced AD).[36] The second was a high quality study of 124 participants in which neuroleptic drug use and grade 2 white matter lesions predicted falls in multivariate analyses, but only participants with AD were included, and there was no control group[37]. The design of our study addressed these issues; to our knowledge, it is the first to examine falls in both AD and non-AD dementias over an adequate follow up period.

Exclusive to our study was the phasic measurement of blood pressure using beat-to-beat recordings, thus enabling detection of both magnitude and timing of blood pressure changes. Symptomatic OH and the length of time blood pressure fell below baseline rather than the magnitude of the drop were predictive of falls. Our group has previously shown an association between intermittent hypotension and white matter lesions,[38] and this resonates with the findings of Horikawa et al. that grade 2 white matter lesions predicted falls in multivariate analyses[37]. White matter lesions can be associated with abnormal gait,[39] and it is possible that intermittent symptomatic hypotension against a background of gait and balance instability exacerbates the likelihood that an older person will fall. A higher level of physical activity was a protective factor in our study. The physical activity scale measured all physical activity rather than wandering, which has been shown in one study to be a predictor of falls in dementia.[40] It is likely that the more active patients were those with fewer problems with mobility, whereas the participants with disturbed mobility restricted their activity because of fear of falling, thus having fewer opportunities to fall.

We believe that randomised multifactorial intervention trials to prevent falls in mild-moderate dementia should now be made a priority. Possible management strategies could include management of the potentially modifiable factors identified in this study; for example, the use of selective serotonin reuptake inhibitors for depression, manipulation of cardiovascular medications, adequate hydration and targeted drug therapies such as fludrocortisone and midodrine for OH. Such a focus would differ from multifactorial interventions in older people without dementia, which prioritise strength and balance exercises that are more difficult for those with impairment of recall to continue following initial intervention. It is possible that encouragement of overall physical activity may be successful in prevention of falls in dementia. However, such an approach may increase opportunities for falling in individuals at risk; similarly, aggressive treatment of motor features in LB dementias might increase activity related opportunities to fall, and also exacerbate OH. There is also a possibility that changes in psychotropic medication might result in side effects such as hypotension or somnolence, which could paradoxically increase the risk of falls. This emphasises the importance of the conduction of randomised controlled trials to ensure that modification of the risk factors identified is the correct strategy.

We conclude that whilst the outcome of future trials are awaited best clinical practice should focus on identification and management of orthostatic hypotension, depression and maintenance of physical activity in individuals who do not have severely impaired gait and balance, whilst bearing in mind the need to monitor patients carefully because of the potential side effects of these changes.
